# Is complementary and alternative medicine (CAM) cost-effective? a systematic review

**DOI:** 10.1186/1472-6882-5-11

**Published:** 2005-06-02

**Authors:** Patricia M Herman, Benjamin M Craig, Opher Caspi

**Affiliations:** 1 Program in Integrative Medicine, University of Arizona, Tucson, Arizona, USA; 2 Department of Pharmacy, University of Arizona, Tucson, Arizona, USA; 3 Recanati Center for Internal Medicine and Research, Rabin Medical Center (Beilinson Campus), Petah Tikva, Israel

## Abstract

**Background:**

Out-of-pocket expenditures of over $34 billion per year in the US are an apparent testament to a widely held belief that complementary and alternative medicine (CAM) therapies have benefits that outweigh their costs. However, regardless of public opinion, there is often little more than anecdotal evidence on the health and economic implications of CAM therapies. The objectives of this study are to present an overview of economic evaluation and to expand upon a previous review to examine the current scope and quality of CAM economic evaluations.

**Methods:**

The data sources used were Medline, AMED, Alt-HealthWatch, and the Complementary and Alternative Medicine Citation Index; January 1999 to October 2004. Papers that reported original data on specific CAM therapies from any form of standard economic analysis were included. Full economic evaluations were subjected to two types of quality review. The first was a 35-item checklist for reporting quality, and the second was a set of four criteria for study quality (randomization, prospective collection of economic data, comparison to usual care, and no blinding).

**Results:**

A total of 56 economic evaluations (39 full evaluations) of CAM were found covering a range of therapies applied to a variety of conditions. The reporting quality of the full evaluations was poor for certain items, but was comparable to the quality found by systematic reviews of economic evaluations in conventional medicine. Regarding study quality, 14 (36%) studies were found to meet all four criteria. These exemplary studies indicate CAM therapies that may be considered cost-effective compared to usual care for various conditions: acupuncture for migraine, manual therapy for neck pain, spa therapy for Parkinson's, self-administered stress management for cancer patients undergoing chemotherapy, pre- and post-operative oral nutritional supplementation for lower gastrointestinal tract surgery, biofeedback for patients with "functional" disorders (eg, irritable bowel syndrome), and guided imagery, relaxation therapy, and potassium-rich diet for cardiac patients.

**Conclusion:**

Whereas the number and quality of economic evaluations of CAM have increased in recent years and more CAM therapies have been shown to be of good value, the majority of CAM therapies still remain to be evaluated.

## Background

Complementary and alternative medicine (CAM) has a reputation for good value among health conscious consumers [1]. In the United States consumers spend over $34 billion per year on CAM therapies [2], dollars spent outside the conventional health care financing system. Such evidence on out-of-pocket expenditures is a testament to the widely held belief that CAM therapies have benefits that outweigh their costs. Regardless of public opinion, there is often little more than anecdotal evidence on the health and economic implications of CAM therapies.

The paucity of outcomes research in CAM has likely depressed access to CAM therapies by impeding their integration into financial mechanisms commonly found in conventional health care. Most US consumers who have health insurance coverage, either through public or private institutions, bear the entire cost of CAM therapies out-of-pocket [3]. Theoretically, CAM therapies seem effective and a good candidate for cost savings because they avoid high technology, offer inexpensive remedies, and harness the power of *vis medicatrix naturae *(the body's natural ability to heal itself). As such, a thorough and external review of economic and health outcomes of CAM is necessary for evidence-based consideration of CAM therapies as a covered expense. That being said, it is also known that affirmative evidence on economic and health outcomes is a necessary, but not sufficient step toward CAM coverage, and not the decision itself. Other factors such as historical demand, political expediency, consumer demand, and practitioner enthusiasm may also be considered in the decision to incorporate CAM into a health insurance policy [1,4,5].

The need for economic evaluations is also growing in conventional healthcare. An increasing number of health plans and hospitals have moved from a simple budgetary focus in formulary decisions to requiring detailed evidence on the economic value of considered therapies relative to alternatives [6,7]. Beyond their use in decisions concerning health insurance coverage, economic outcomes of both CAM and conventional therapies also influence health policy, justify licensure of practitioners, inform industry investment decisions, provide general evidence to consumers about potential economic benefits, and can guide future research efforts through identifying decision-critical parameters for additional research [8,9].

In their systematic review of CAM economic evaluations, White and Ernst [4] identified 34 economic evaluations of CAM conducted between 1987 and 1999; only eleven of which were full economic evaluations (ie, compared both economic and health outcomes between two or more alternatives) [10]. Quality was evaluated by noting whether cost data were collected prospectively and whether comparison groups were comparable – ie, assigned randomly. Unfortunately, their search strategy included the term "alternative medicine" but not "complementary medicine." Therefore, all single therapy studies in their review are of CAM therapies that are usually used as substitutes (alternatives) to conventional care (eg, acupuncture, homeopathy, and spinal manipulation). No studies of complementary therapies (those used in conjunction with conventional care) were included, despite the use of the term "complementary" in their conclusion that spinal manipulative therapy may have benefits for back pain, but "there was a paucity of rigorous studies that could provide conclusive evidence of differences in costs and outcomes between other complementary therapies and orthodox medicine [4]."

The objectives of this paper are: 1) to introduce concepts commonly applied in economic evaluations of health technologies (often called technology assessment) so that practitioners and CAM users can translate and benefit from published evidence; and 2) present a systematic review of the current scope and quality of economic evaluations of CAM. We begin with an overview of economic evaluation, including didactic examples from the CAM economic literature to help clarify the concepts presented. Readers familiar with this type of analysis can skip this section and proceed directly to the methods section.

In our systematic review we expand upon and update the initial review by White and Ernst. We evaluate study quality in more detail, using both additional study design criteria and quality of reporting criteria, and present a summary of the results from exemplary studies. While their review was the first of its kind, economic evaluations in the CAM literature have improved greatly in the last five years. We end the paper with a description of the attributes of CAM that make economic evaluation challenging and how these issues may be addressed. We hope that practitioners' interest in economic evaluation will continue to grow, leading to greater incorporation of this research into CAM trials.

### What is an economic evaluation?

An economic evaluation is a comparison of outcomes among alternative ways of achieving common objectives. These analyses are conducted according to explicit, systematic, and consistent criteria, and take into account both the positive and negative consequences of each alternative. Consequences may include economic, clinical, and humanistic outcomes, known as the ECHO model [11]. Economic outcomes represent the consumption and production of resources and their monetary value from the perspective of a decision maker. Clinical outcomes are medical events that are professionally meaningful. Humanistic outcomes are a broad category of intangible personal attributes, typically collected through self-report. Humanistic outcomes include quality of life characteristics such as sense of safety, physical comfort, enjoyment, meaningful activity, relationships, functional competence, dignity, privacy, individuality, autonomy, and spiritual well-being. Conventionally, clinical and humanistic outcomes are considered health outcomes, and we follow this convention for the remainder of the article.

There are several forms of economic evaluations that can be performed (cost-effectiveness analysis being only one of these) and each differs based on the selection and measurement of health outcomes. The perspective (or point of view) taken for the analysis also influences the selection and measurement of consequences, because not all outcomes are important to all decision makers. Generally, there are three perspectives for economic analysis: individual (eg, patient), institutional (eg, health maintenance organization), or societal. The societal perspective accumulates all outcomes, while individual and institutional analyses are more selective. Regardless of perspective, the objective of an economic evaluation is to provide information on consequences relating to alternatives faced by a decision maker.

The most basic form of economic evaluation is a table that lists the individual economic and health outcomes of alternative interventions. This table is known as a cost-consequence study. Cost-identification studies and cost-minimization analyses only address economic outcomes and are discussed below in that section. The remaining forms of economic evaluations summarize economic and health outcomes into a single result (Table 1).

**Table 1:** Three forms of full economic evaluations

	Cost-benefit Analysis (CBA)	Cost-effectiveness Analysis (CEA)	Cost-utility Analysis (CUA)
Number of Health Outcomes	Multiple outcomes	One outcome	Multiple outcomes
Unit of Health Outcomes	Summary measure in monetary units (eg, US dollars)	Natural units (eg, reduction in number of hot flashes)	Summary measure in quality of life units (eg, quality-adjusted life-years, QALY)
Results	Net benefits (B_1 _+ B_2 _- C_1 _- C_2_)	Cost-effectiveness ratio* (C_1 _- C_2_) /(E_1 _- E_2_)	Cost-utility ratio* (C_1 _- C_2_) /(QALY_1 _- QALY_2_)

* Results are calculated when both the costs and the effects (health outcomes) of one therapy are higher than those of another. When the costs are lower and the effects are higher for one therapy, it is said to dominate the alternative (and the alternative is said to be dominated) and no ratio is presented. C_1 _= total costs of alternative 1; C_2 _= total costs of alternative 2; B_1 _= monetary value of health outcomes of alternative 1; B_2 _= monetary value of health outcomes of alternative 2; E_1 _= health effects of alternative 1; E_2 _= health effects of alternative 2; QALY_1 _= quality-adjusted life-years of alternative 1; QALY_2 _= quality-adjusted life-years of alternative 2.

The advantages of performing cost-benefit and cost-utility analyses are that multiple outcomes are summarized into a single unit, either monetary units such as dollars (CBA) or QALYs (CUA) and that therapies with different sets of health outcomes can be compared based on the differences in the summary measures. Cost-benefit analysis has the additional benefit of directly indicating whether the therapy pays for itself.

The disadvantages of CBA and CUA come from the techniques required to produce a summary measure. Cost-benefit analysis requires putting a monetary value on all health outcomes (and ultimately on life), and cost-utility analysis assigns value to health outcomes based on their contribution to quality of life under the presumption of population-based preferences. An extensive literature addresses the methodological and theoretical issues involved in the construction of these summary measures. The process usually occurs in two steps. In the first step, health outcomes of the intervention are measured, and in the second the outcomes are valued in summary units and aggregated. Cost-benefit analyses often assess the monetary value of health outcomes based on willingness-to-pay using a technique called conjoint analysis [12-14]. Willingness-to-pay inherently places a lower values of life on individuals with low income, because they can not pay what they do not have. Cost-utility analyses have multiple methods to place quality of life values on health outcomes, also known as social tariffs. Summary measures of quality of life may not be sensitive enough to pick up short-term changes such as for acute conditions and will not pick up specific clinical outcomes like blood pressure control [15]. Examples of instruments used to capture these general health states include the EuroQoL (EQ-5D) [16] and the Health Utilities Index [17].

Cost-effectiveness analysis (CEA) is the current standard in the literature, and has the most straight forward interpretation. Under CEA, therapies useful for a specific disease or condition can be directly compared using a metric of effectiveness relevant to that condition, such as blood pressure control. Although these types of analyses do not allow a summary of multiple outcomes they tend to respond well to the most urgent questions, such as how much would it cost to reduce the number of gestational diabetes cases by 10%? Clearly, a reduction in gestational diabetes cases has measurable implications in quality of life and economic units, but the creation of a summary measure is not necessary to address the decision maker's question.

No matter the approach taken, it is recommended that the estimated outcomes (economic, clinical and humanistic) of health care alternatives used in economic evaluation are best estimated in pragmatic clinical trials that directly and realistically compare the therapies of interest [10]. Rarely are the results of placebo-controlled trials appropriate [1,4,18-20]. Also, since many CAM therapies target chronic disease, it is important that the study period be long enough to capture the full benefits and costs of each therapy, and that future costs and benefits be discounted to the present for comparison. Finally, all economic evaluations should include some type of sensitivity analysis to test the robustness of results to the various assumptions made [1,20,21].

### What are economic outcomes?

Economic outcomes are the net bundle of resources forgone due to an intervention valued at the opportunity cost of those resources (the value of their next best use or "opportunity"). Since the cost of a therapy differs depending on whether you are a patient, a health plan, or a health care provider, the economic outcomes (ie, costs) of each therapy depend on the perspective of the study. Studies that only measure the economic outcomes of interventions are known as cost-identification studies. A study that describes the economic and health outcomes of a single therapy can also be called a cost-identification study. These studies inform full economic evaluations. That is, they provide the data needed to better design future studies that consider both the economic and health outcomes of two or more alternative therapies. A cost-minimization analysis (CMA) explicitly assumes equivalence in health outcome among alternative therapies, and examines only economic outcomes. In practice, it appears the same as a cost-identification study, but under the assumption of equivalence, a CMA is a full economic evaluation.

Table 2 has been summarized from other references [1,20,22] and gives a list of the types of economic outcomes and the perspective of analysis where each is considered. Note that these types of economic outcomes should be inclusive of both the full costs of the therapy and of any treatment for adverse effects, which can be expensive. In economic evaluations, the safety of a therapy is addressed through accounting for the cost of treating these adverse events as well as through their impact on clinical and quality of life outcomes.

**Table 2:** Economic outcomes to include in economic evaluation

Type of Cost	Examples	Perspectives in Which This Cost is Included
Direct costs: Medical	Intervention costs: Practitioner fees Diagnostic costs Therapy costs Service costs: Facilities and equipment, including hospitalization or clinic/office costs Ancillary staff	Portion paid by health plan included in institutional perspective Portion paid by patient included in individual perspective All included in societal perspective
Direct costs: Non-medical	Transportation costs Time off work for appointments/hospitalization	Usually all paid by the patient, so often included in individual perspective All included in societal perspective
Indirect costs	Lost work productivity during recuperation Lost leisure time Child care costs Costs to care givers	Usually all paid by the patient, so often included in individual perspective All included in societal perspective
Intangible costs	Pain Suffering Grief	Not usually included as costs; instead, may be included in humanistic outcomes in cost-utility analysis

Summarized from similar tables in other references [1, 20, 22].

It is recommended that economic outcome data are best collected prospectively as part of a pragmatic clinical trial [1,4,19,20]. Inclusion and exclusion criteria for cost data should be established in the protocol, as for clinical outcome measurements, but provision must be made to add extra categories of costs which only become apparent after the trial has commenced [1,20]. Many studies try to collect cost data retrospectively, often after a therapy has shown clinical effectiveness. However, retrospective data collection is seldom fertile, adapted, or exhaustive, and it is subject to bias [18,20].

### Examples of the different forms of economic evaluations of CAM

Our systematic review of the CAM economic evaluation literature (presented below) revealed no cost-consequence studies and no cost-benefit analyses. However, we did find examples of a cost-identification study, cost-minimization analysis, cost-effectiveness analysis, and cost-utility analysis. These examples are presented below.

#### Cost-identification study

Frenkel and Hermoni, 2002 [23], performed a retrospective comparison of medication consumption costs from computerized medication charts three months before and three months after a homeopathic intervention for atopic and allergic disorders. The review was performed on 48 consecutive self-referred patients in one clinic over one year with a diagnosis of an atopic condition who agreed to a classical homeopathic treatment in addition to usual conventional care. Of the 31 medication users (prescription and non-prescription allergy-related medications) before the intervention, 27 reduced their use, two increased their use, and two had their medication level unchanged after the intervention. Of the 17 who had not used medication before the intervention, 4 began medication after the intervention. There was an average drop in 3-month medication costs after homeopathy of $14 (1998 US$) or 54% per person.

#### Cost-minimization analysis

Herron and Hillis, 2000 [24], retrospectively compared government payments to physicians for 1418 Quebec health insurance enrollees who practiced the Transcendental Meditation (TM) to payments for 1418 randomly selected and matched enrollees who did not. Long term health outcomes were assumed to be equal for both groups. Before starting meditation, the groups were similar in the yearly rate of increase in payments. After starting TM, annual physician payments for the meditation group declined 1 to 2% per year, while those for the non-TM group increased annually over the six year period. The difference in the annual change in payments was statistically significant at a rate between 5 and 13% per year.

#### Cost-effectiveness analysis

Franzosi et al, 2001 [25], prospectively gathered health and economic outcomes during the 3.5 year follow-up period of a large randomized open-label study (n = 5664) of omega-3 polyunsaturated fatty acids (n-3 PUFA) as secondary prevention for patients with recent myocardial infarction. The perspective was that of a third-party payer; accordingly only direct health care costs (hospital admissions, laboratory and diagnostic tests, and medications) were considered. The incremental number of life-years saved by n-3 PUFA treatment over the 3.5 years (discounted at 5%) was 0.0332 per patient. The incremental cost discounted over the same period was 817€ per patient. Therefore, the incremental cost-effectiveness ratio is 24,603€ (approximately $25, 415 in 1999 US$ [26]) per life-year saved.

#### Cost-utility analysis

Korthals-de Bos et al, 2003 [27], performed an economic evaluation alongside a randomized controlled trial to compare manual therapy, physiotherapy, and care by a general practitioner for neck pain. The study used the societal perspective and collected direct and indirect costs (including hours of help from family and friends, and hours of absenteeism from work or other activities) through the use of cost diaries kept by patients over one year. Data on each patient's overall health state were gathered at baseline and at one year using a survey instrument called the EuroQoL [16]. The utility of these health states were then calculated by using "society's" preferences for each of those health states. Society's preferences were estimated from a sample of the general population by the developers of the EuroQoL instrument. Using the comparison of manual therapy to general practitioner care, manual therapy had a lower one-year cost ($402, US$) than general practitioner care ($1241). The QALYs were 0.82 for manual therapy and 0.77 for general practitioner care. Since the costs were lower and the QALYs higher for manual therapy as compared to usual care, manual therapy is said to dominate general practitioner care and no cost-utility ratio is calculated.

## Methods

The National Center for Complementary and Alternative Medicine (NCCAM) defines CAM as "a group of diverse medical and health care systems, practices, and products that are not presently considered to be part of conventional medicine [28]." We further defined CAM as including only those therapies that could be prescribed (or recommended) and/or performed by a CAM practitioner who does not also have a conventional medical license (eg, doctor of medicine – MD, or doctor of osteopathy – DO). Therefore, we did not include therapies such as chemotherapy regimens nor therapies requiring surgical implantation (such as neuroreflexotherapy [29]) as CAM therapies even though these therapies do appear in searches using the keywords complementary and/or alternative medicine. We also did not include well-accepted vitamin and mineral supplementation therapies such as calcium and vitamin D for osteoporosis, niacin for dyslipidemia, and vitamin B12 and folic acid for homocysteine reduction.

### Search strategy

We searched the following electronic databases from January 1999 to October 2004: Medline, AMED, Alt-HealthWatch, and the Complementary and Alternative Medicine Citation Index via NCCAM and the National Library of Medicine (NLM). Searching was restricted to English language journals and human studies with the keywords: complementary medicine or alternative medicine, and costs or cost analysis or cost-benefit or cost-effective or economic analysis or economic evaluation.

We removed duplicates from the search results and selected papers that reported original data on specific CAM therapies from any form of standard economic analysis, analysis of costs, or economic modeling. Studies were then excluded if they were cited in the White and Ernst review [4], or if they were case studies or case series of five or fewer subjects.

### Data analysis

The following data were extracted from each of the included studies: full citation information (author(s), date, title, journal, etc), form of economic evaluation (stated or inferred), the therapies being compared and whether the CAM therapies were being used in addition to usual care (complementary) or instead of usual care (alternative), the perspective of analysis (stated or inferred), the study design, the sample size, and summary results.

The studies were categorized as either full economic evaluations (defined as a comparison between two or more alternatives and considering both costs and consequences [10]) or partial economic evaluations (those studies that did not contain a comparison, or only addressed costs). Studies that estimated resource utilization were included as full economic evaluations even if resources were not valued.

We captured data on quality of the full economic evaluations using two approaches. The first approach was to gather from each study the data needed to assess quality according to a 35-item checklist developed by the *BMJ *Economic Evaluation Working Party [30]. This checklist was developed to improve the quality of published economic evaluations, and was chosen because it is thorough, and entails an objective assessment of whether essential components of an economic evaluation are reported in the article. Therefore, the checklist is mainly a measure of reporting quality and not necessarily of study quality. We also report available results from several other general reviews of economic evaluations of conventional therapies that use this checklist for comparison.

As the purpose of economic evaluations is to inform clinical practice and health policy decisions, the best evaluations are timely and use the best data available at the time [10]. On the other hand, an evaluation is only as good as the data upon which it is based. It has been suggested that the ideal situation for data collection is to collect economic data along side health outcomes in a randomized pragmatic trial [10]. Pragmatic trials offer a compromise between the goals of internal and external validity. To assess study quality, we went beyond White and Ernst's [4] criteria of randomization (to reduce bias by creating comparable groups) and prospective collection of economic outcome data (to ensure all costs are captured) to include two additional indicators of whether a pragmatic (effectiveness or "real world") rather than efficacy trial was conducted. The first is that the comparison group was usual care, and the second was that the study was not blinded and not mandatory – ie, that physicians and patients could react realistically to the therapy [10]. These criteria relate to the external validity or generalizability of the study. Other indicators of a study's generalizability, such as the determination of whether study participants could be assumed to represent a normal case load, were not used as they required detailed knowledge as to the appropriateness of the inclusion and exclusion criteria for each condition studied – a level of expertise not held by the study's authors.

Based on the study quality criteria, we report summarized results of the exemplary studies – ie, those meeting all four study quality criteria. If the health outcomes for one therapy are better than that of its alternative and the economic outcomes are better or equal (lower or equal costs), that therapy is said to dominate (be clearly better than) its alternative. This is also the case if both therapies have equal health outcomes and one has lower costs. In all other cases, the decision maker must elect whether the increase (loss) in health benefits is worth the increase (savings) in cost.

## Results

The database search rendered 1765 potential studies to screen. Application of inclusion and exclusion criteria reduced the list to 56 economic evaluations [23-25,27,31-82]. The therapies compared, study design employed, sample size used, and a summary of study results are provided for each study in an appendix [see Additional file 1]. The list contains 39 full evaluations and 17 partial evaluations. The evaluations cover a range of CAM therapies applied to a variety of conditions (Table 3). Some therapies, such as acupuncture, homeopathy, and manual therapy, were studied mainly as alternative therapies (ie, as substitutes or alternatives for conventional care). Other therapies, such as guided imagery, were studied as complementary therapies (ie, used in addition to conventional care).

**Table 3:** Types of complementary and alternative medicine (CAM) therapies studied for various conditions (full/partial economic evaluations)

	Acupuncture	Homeopathy	Manual therapy	Spa therapy	Mind-body therapy	Hypnosis	Botanical medicine	Nutritional supplements	Diet	Biofeedback	Hyperbaric oxygen therapy	Miscellaneous*	TOTALS†
Populations with mixed conditions‡		3/2			2/1			0/1				0/1	10
Back, neck, and/or leg pain	1/0		5/0				1/0					1/0	8
Surgery						2/1		2/0					5
Cardiac patients					2/0			1/0	1/0				4
Rheumatic disorders		0/1		1/0						1/0			3
Epilepsy									0/3				3
General costs	0/1		0/2										3
Allergy		0/1										1/0	2
Cancer chemotherapy					2/0								2
Diabetic ulcers											2/0		2
Dyspepsia	1/0	1/0											2
EENT in children		1/1											2
Headache/migraine	2/0												2
Midwifery/obstetrics										1/0		0/1	2
Miscellaneous§		1/0		2/0	1/0		1/0	1/2	2/0				10
TOTALS†	5	11	7	3	8	3	2	7	6	2	2	4	60

EENT = Eye, ear, nose, and throat conditions

* Miscellaneous CAM therapies include: multivitamins, shoe orthoses, electrodermal screening, and aromatherapy.

† Some studies compared more than one CAM therapy. Therefore, totals exceed the number of studies found.

‡ Populations with mixed conditions include: patients with chronic disease, patients at one general practice (4 studies), long-term care workers, persons in Quebec health system, inner city children, and older adults (2 studies).

§Miscellaneous conditions include: anxiety, Parkinson's, psoriasis, uterine fibroids, urinary tract infection, macular degeneration, severe burn, AIDS, obesity, and hypertension.

### Reporting quality checklist

Table 4 shows the results of the application of the *BMJ *35-item quality checklist [30] to the 39 full economic evaluations. For comparison, Table 4 also contains comparable results from systematic reviews in conventional medicine [6,83,84].

**Table 4:** Reporting quality of complementary and alternative medicine (CAM) economic evaluations and comparable results of similar reviews in conventional medicine

Items from the *BMJ *Checklist [30] (Indented items apply only to a subset of studies)	Review of CAM Studies N (%)	Reviews of Conventional Medicine Studies N (%)
**Study design**		
(1) The research question is stated	39 (74)	43 (16)*
(2) The economic importance of the research question is stated	39 (51)	
(3) The perspective of the analysis is stated	39 (33)	228 (52)†
(4) The rationale for choosing the alternatives is stated	39 (69)	
(5) The alternatives being compared are clearly described	39 (74)	228 (83)†
(6) The form of economic evaluation used is stated	39 (49)	
(7) The choice of form of economic evaluation is justified	39 (3)	43 (7)*
**Data collection**		
(8) The source(s) of effectiveness estimates are stated	38 (100)	
(9) Details of the effectiveness study are given	36 (94)	
or (10) Details of the review or meta-analysis are given	2 (50)	
(11) Primary outcome measures are clearly stated	39 (95)	
(12) Methods to value health states are stated	4 (100)	228 (75)† 43 (79)‡
(13) Details of the subjects from which values were obtained are given	4 (25)	228 (76)† 43 (46)‡
(14) Productivity changes are reported separately	8 (88)	
(15) The relevance of productivity changes is discussed	8 (25)	
(16) Quantities of resources are reported separately from unit costs	39 (67)	43 (19)‡
(17) Methods for the estimation of quantities and unit costs are described	39 (67)	
(18) Currency and year are recorded	39 (41)	228 (68)†
(19) Details of adjustments for inflation or currency conversion are given	39 (21)	43 (21)*
(20) Details of any model used are given	3 (100)	
(21) The choice of the model and its key parameters are justified	3 (100)	
**Analysis and interpretation of results**		
(22) Time horizon of costs and benefits is stated	39 (100)	
(23) The discount rate is stated	4 (50)	228 (65)†
(24) The choice of discount rate is justified	4 (25)	43 (16)* 34 (21)‡
(25) An explanation is given if costs and benefits not discounted	4 (50)	8 (12)‡
(26) Details of statistical tests and confidence intervals are given for stochastic data	38 (87)	
(27) The approach to sensitivity analysis is given	5 (100)	43 (2)*
(28) The choice of variables for sensitivity analysis is justified	5 (40)	39 (79)‡
(29) The ranges over which variables are varied are stated	5 (100)	228 (57)† 38 (66)‡
(30) Relevant alternatives are compared	39 (36)	228 (57)†
(31) Incremental analysis is reported	13 (54)	228 (46)†
(32) Major outcomes are presented disaggregated and aggregated	39 (85)	
(33) The answer to the study question is given	39 (69)	
(34) Conclusions follow from the data reported	39 (100)	
(35) Conclusions are accompanied by the appropriate caveats	39 (67)	228 (84)†

* Comparable estimates available from Jefferson et al, 1998 [83].

† Comparable estimates available from Neumann, 2004 [6], a systematic review of cost-utility analyses.

‡ Comparable estimates available from Gerard et al, 2000 [84], a systematic review of cost-utility analyses.

#### Study design

These checklist items indicate whether essential components of the study design were reported. About half the studies stated the form of the economic evaluation, however, several were stated incorrectly and only one justified the form chosen. The bulk of the studies presented cost-effectiveness analyses (36 or 92%), five presented cost-utility analyses, and one was a cost-minimization study [24]. Only one-third of studies stated the perspective of the analysis, however, it could be determined from the costs included for all studies. Ten used a societal perspective, and the majority (33 or 85%) used some sort of institutional perspective (eg, health insurance company or hospital). Note that the totals by form and perspective add to more than 39. This is because individual studies can include analyses using more than one form of economic evaluation and can report costs from more than one perspective.

#### Data collection

These checklist items relate to the presence of information essential to the generalizability of study results. All studies that included health outcomes (ie, all except the one cost minimization study [24]) reported the source of their effectiveness estimates. In 36 of the 38 cases the source was a single study, often the economic evaluation itself. The two other studies were modeling studies [49,78] where reviews were used as the source of effectiveness estimates. Items 12 and 13 are appropriate for cost-utility analyses (where health states are valued in terms of utility) and there were four such studies [27,35,51,78], only one of which gave details on the subjects from whom the valuations were obtained [35]. Productivity changes (items 14 and 15) are appropriate for studies using the societal perspective. Eight studies included the costs of changes in productivity from improvement in back or leg pain [47,82], neck pain [27], migraine [32], anxiety [44], ankylosing spondilitis [51], psoriasis [49], and children's rhinopharyngitis [41]. All but one [49] reported these amounts separate from total costs. However, few discussed the relevance to the study of productivity changes.

About two-thirds of studies reported resource use quantities separate from unit costs, or described the methods used to estimate both quantities and unit costs. Whereas, almost all reported the currency used, only a minority (16 or 41%) reported the currency year. A smaller number reported the details of adjustments for inflation or currency conversion, but this was not often required in studies collecting and reporting data in the same year and currency. Models (one decision tree model [78] and two multiplicative-type or impact [49,57] models) were used in three studies and in all cases the details of the model were given and justified.

#### Analysis and interpretation of results

All studies stated the time horizon for costs and benefits and most (35 or 90%) reported a time horizon of one year or less. Items 23 through 25 apply only to the four remaining studies with time horizons longer than one year. The discount rate is reported in two of these studies (one with a time horizon of 42 months [25] and the other that included a 12-year projection [78]), but only one justified the choice of discount rate [78]. Two studies gave an explanation for why they did not discount costs and benefits, however, neither needed to – one had a one-year time horizon [35] and the other stated its time horizon as one course of chemotherapy [55]. Five studies performed sensitivity analyses [25,27,35,51,78]. In all cases the approach and the range of variables tested were stated, but the choice of variables to test was only justified in two cases [35,51].

In about one-third of studies there was some comparison of study results to that of other studies. In most cases this was done as a simple statement noting that the results were either similar, or that they were dissimilar and that this might be because of differences in study design. Incremental cost-effectiveness or cost-utility ratios are usually only required when one therapy offers clearly better health outcomes than the other, but at a higher cost. In the 13 studies where this was the case over one-half reported incremental analyses. In most cases the major outcomes of the studies were shown disaggregated, and the study question was answered. We did require that a proper research question be stated (see the answers to item 1) for it to be answered. In all cases, we felt that the conclusions followed the data, but in about one-third of cases the conclusions were not presented with the appropriate caveats. For example, if a study did not explicitly discuss its limitations, it was not included as meeting the last item.

### Measures of study quality

Twenty-seven studies (69%) gathered cost data prospectively and 21 (54%) used randomly assigned comparison groups. In 32 studies (82%) the physicians and patients were not blinded to the treatment received and participation was not mandatory (a worksite intervention [57]), and therapies were compared to usual care in 34 (87%) of studies. Fourteen studies [25,27,32,34,35,50,51,53-55,68,74,76,82] met all study quality criteria, and a summary of their results is shown in Table 5.

**Table 5:** Summary of the results of complementary and alternative medicine (CAM) economic evaluations with exemplary study quality

	CAM Therapy Compared to Usual Care*	Patient Population	Form of Economic Evaluation	Health Effects of CAM Compared to Usual Care†	Cost of CAM Compared to Usual Care†
Liguori et al, 2000 [32]	Acupuncture	Patients with migraine	CEA	**Better**	**Lower‡**
Wonderling et al, 2004 [35]	Acupuncture	Patients with chronic headache	CUA	Better	Higher‡
Paterson et al, 2003 [34]	Acupuncture	Patients with dyspepsia	CEA	Similar	Similar
	Homeopathy		CEA	Similar	Similar
Korthals-de Bos et al, 2003 [27]	Manual therapy	Patients with neck pain	CEA CUA	**Better** **Similar**	**Lower¶**
Brefel-Courbon et al, 2003 [50]	Spa therapy	Patients with Parkinson's disease	CEA	**Similar**	**Lower**
Van Tubergen et al, 2002 [51]	Combined spa-exercise therapy	Patients with ankylosing spondylitis	CEA CUA	Better Better	Higher¶
Tusek et al, 1999 [53]	Complementary guided imagery	Cardiac surgery patients	CEA	**Better**	**Lower**
van Dixhoorn and Duivenvoorden, 1999 [54]	Complementary relaxation therapy	Patients with previous myocardial infarction	CEA	**Better**	**Lower**
Jacobsen et al, 2002 [55]	Complementary professionally-administered stress management training	Cancer patients undergoing chemotherapy	CEA	Similar	Higher‡
	Complementary self-administered stress management training		CEA	**Better**	**Lower‡**
Franzosi et al, 2001 [25]	Complementary omega-3 polyunsaturated fatty acids	Patients with recent myocardial infarction	CEA	Better	Higher
Smedley et al, 2004 [68]	Complementary preoperative and post operative oral nutritional supplementation	Patients undergoing lower gastrointestinal tract surgery	CEA	**Better**	**Similar**
Norris et al, 2004 [56]	Potassium-rich diet	Postoperative cardiac patients	CEA	**Similar**	**Lower**
Ryan and Gevirtz, 2004 [76]	Biofeedback-based psychophysiological treatment	Patients with "functional" disorders (e.g., irritable bowel syndrome)	CEA	**Better**	**Lower**
Larsen et al, 2002 [82]	Complementary custom-made biomechanical shoe orthoses	Recent military conscripts	CEA	Better	Higher

**Bold **entries indicate that the CAM therapy was shown to be clearly superior to (dominate) usual care.

CEA = cost-effectiveness analysis; CUA = cost-utility analysis

* The use of the term "complementary" in this column indicates CAM therapies used in addition to usual care.

† If tests of statistical significance were performed, costs must be significantly higher or lower (and health effects significantly better or worse), or they were considered "similar."

‡ This study used both a societal and an institutional perspective, and the results were in the same direction.

¶This study used a societal perspective only. All other studies used an institutional perspective only.

## Discussion

The number of economic evaluations of CAM has increased in recent years, even if we only count full evaluations of alternative therapies. Study quality has also increased, and although reporting quality can use improvement, it is on the whole similar to that seen in economic evaluations of conventional medicine. Nevertheless, there are still too few good quality evaluations to draw many conclusions about the cost-effectiveness of specific CAM therapies for particular conditions.

### Potential reasons for paucity

A possible explanation for the paucity of studies is that there may be less of an incentive to perform economic evaluations of CAM. Consumers are already spending a large amount of their disposable income on CAM without formal proof of effectiveness or cost effectiveness. Economic evaluations are typically required for the incorporation of therapies under traditional financing mechanisms and for adjustment of coverage under these mechanisms. Therefore, the market for economic evaluation in CAM may be small due to reduced involvement of third-party payers in CAM financing.

Some CAM practitioners do not see the need for economic evaluations. An interesting study by Kelner et al [85] asked chiropractors, homeopaths, and Reiki practitioners about the need to demonstrate the effectiveness, safety and cost effectiveness of their therapies. The chiropractors agreed that high quality economic evaluations are essential to their practice, but Reiki practitioners could see no reason for this research, and the homeopaths were divided on these issues. There may be good reason why some practitioners resist economic evaluation. If studies are performed that show economic benefit of CAM therapies, third party reimbursement may follow which could reduce practitioner autonomy. Coverage may also be restricted to the standardized forms of botanical medicines, nutritional supplements, or protocols used in the studies [86]. This could dramatically change how CAM is practiced by decreasing the use of multidimensional multicomponent interventions, by institutionalizing care into conventional health care systems, and by limiting the individualization of care.

### Relative quality of evaluations

The reporting quality was poor for certain items, but was comparable to the quality found by systematic reviews of economic evaluations in conventional medicine [6,83,84]. Although the *BMJ *checklist was mostly objective (ie, required the least amount of judgment compared to the other checklists available), a fair amount of interpretation was still required for many items. For example, in our review we interpreted Item 1 as whether the study stated either a specific research question or study objectives in terms of economic and health outcomes. Three-quarters of the full economic evaluations of CAM met this criterion. However, in Jefferson et al, 1998 [83], only 16% of the 43 economic evaluations of conventional medicine reviewed where identified as fulfilling Item 1. It is likely that Jefferson et al took a more restrictive interpretation of this quality criterion.

Several studies have shown that at least some aspects of quality in economic evaluations improve over time [6,87]. Our findings suggest a trend of quality improvement in these studies in CAM. We found that 69% (27 of 39) of the cohort of full economic evaluations collected cost data prospectively as compared to 45% (5 of 11) in White and Ernst's review. Similarly, we found that 54% (21 of 39) of our studies used randomization to create the comparison groups as compared to 45% (5 of 11) in White and Ernst's review.

We found that 14 (36%) of full economic evaluations met all four study quality criteria and were identified as exemplars. However, the evidence from these criteria must be interpreted cautiously; meeting all study quality criteria does not guarantee an adequate study design. Some aspects of what makes a good pragmatic trial could not be judged by what was reported. For example, pragmatic trials enroll patients typical of normal caseload in typical settings with average physicians following them under routine conditions [10]. Judgments as to whether these criteria were met were not possible because of vague reporting. It is also not generally agreed across all health economists that a pragmatic trial, even a well-designed one, can fully represent the real world of health care. These economists advocate for the collection of cost data using an observational study design.

A study may also be of "poor" quality because it applied the CAM therapy inappropriately. This can happen when a study is designed by researchers not familiar with a therapy. In response to this problem researchers and practitioners of several CAM therapies have begun development of standards for research and reporting. Reporting standards do not guarantee that the therapy was used appropriately, but they at least allow determination of what was done. One such set of reporting standards are the STRICTA recommendations for acupuncture [88]. Of the four full evaluations of acupuncture, two (one of which was included in Table 5[32]) met STRICTA reporting standards. As these types of guidelines are not yet available for all CAM therapies, we did not assess whether CAM therapies were applied appropriately in the studies reviewed.

### Cost-effectiveness of CAM

The exemplary studies summarized in Table 5 indicate that a number of CAM therapies may be considered cost-effective compared to usual care for a number of conditions: acupuncture for migraine, manual therapy for neck pain, spa therapy for Parkinson's, complementary guided imagery for cardiac surgery patients, complementary relaxation therapy for patients with previous myocardial infarction, complementary self-administered stress management for cancer patients undergoing chemotherapy, complementary pre- and post-operative oral nutritional supplementation for lower gastrointestinal tract surgery, potassium-rich diet (rather than potassium supplements) for postoperative cardiac patients, and biofeedback for patients with "functional" disorders such as irritable bowel syndrome. Acupuncture and homeopathy were both found to be equivalent in terms of effects and costs to usual care for dyspepsia. The attractiveness of the other CAM therapies shown in Table 5 depends on whether the increased health benefits are worth the additional cost, or whether other aspects of the therapy make them attractive, such as patient preference. Only one of the studies summarized in Table 5 reported results of a CAM therapy being dominated by (clearly inferior to) usual care. The use of professionally-administered stress management for cancer patients undergoing chemotherapy was shown to have higher costs, but no additional health benefits over usual care. It is important for CAM that this contradictory evidence is also known for best clinical practice and the efficient use of CAM resources.

On the surface one might expect that therapies that substitute for usual care (alternative medicine) would be much more likely to be cost effective. In this sample of exemplar studies, of the nine study comparisons where CAM therapies were shown to be superior to usual care (better effects and lower costs, similar effects and lower costs, or better effects and similar costs), four were studies of complementary therapies. Therefore, there is evidence that even though complementary therapies are given in addition to usual care, they can improve clinical outcomes without increasing costs.

### Issues specific to the economic evaluation of CAM

In many ways the economic evaluation of CAM therapies is similar to that of conventional medicine. However, there are a number of issues specific to CAM that must be considered. These issues can roughly be divided into three groups: those involved with the impact of economic evaluation on CAM in general, those involving the estimation of health outcomes (ie, issues involved with estimating the efficacy or effectiveness of CAM), and those specific to CAM's economic and humanistic outcomes. The first group of issues has already been addressed above under the potential reasons for paucity.

The methodological challenges involved in determining the clinical effectiveness of CAM have been discussed at length in a number of papers. These include the appropriateness of population-based studies when individualized treatments are used and individualized outcomes are expected [89-91], reductionist focus on one therapy for one outcome when that therapy comes from a holistic healing system [92-94], the difficulties with blinding when no appropriate placebo is available [94,95], and the requirement for randomization when most CAM users have strong preferences for their therapy of choice and will often either refuse to be randomized, or will bypass the randomization if it is not to their liking [94]. These challenges are relevant to economic evaluations since they are dependent on effectiveness studies for health outcomes. Also since humanistic and economic outcomes are ideally measured alongside health outcomes in the same trials [1,4,19,20], the challenges above are also relevant to their measurement.

However, there are several additional issues specific to CAM humanistic and economic outcome measurement which must be considered. First, although CAM therapies can be used to treat acute conditions, they are more commonly used to treat chronic disease, to prevent future disease (risk reduction), and to optimize health and well-being. Using CAM for those indications requires that long term studies be performed [96]. However, there are a number of challenges inherent in long term studies in addition to the increase in cost (eg, increased loss to follow-up through patient attrition) [97]. In our systematic review we found only two clinical trials that followed patients prospectively longer than one year: a five-year study of relaxation therapy for patients with a previous myocardial infarction [54], and a 3.5-year study of n-3 PUFA as secondary prevention for patients with previous myocardial infarction [25].

Economic evaluations in CAM must recognize that the process of healthcare itself can be effective for patients. Attributes of the process of using CAM that may have value include patient empowerment, the operationalization of patient preference for a particular type of intervention, the length and process of the consultation, and still having treatment options open when other medical approaches have failed [4,98]. Therefore, economic evaluation of CAM needs to measure and include this value where appropriate.

Optimizing health, maximizing wellness, and enhancing well-being are patient-centered outcomes – ones that by definition require subjective measurement [99]. Economic evaluation of CAM must include appropriate measurement of these humanistic outcomes to account for the full value of CAM therapies. Our systematic review found five studies where humanistic outcomes were captured. The more well-known instruments used to measure health status in these studies included the SF-6D [35] and the EuroQoL (EQ-5D), and health status was translated into quality of life units using population-based preferences [27,51]. Sensitivity of these instruments to the changes in quality of life is an important concern for the evaluation of CAM therapies. Although the use of the EuroQoL for manual therapy for neck pain [27] resulted in a statistically insignificant change in quality of life, two other studies demonstrated small, but statistically significant differences in quality of life using the SF-6D for acupuncture for chronic headache [35], and using the EuroQoL for spa therapy for ankylosing spondylitis [51]. Therefore, it is possible to measure a change in humanistic outcomes for CAM therapies with these instruments.

The collection of economic outcome data is complicated by that fact that in the United States and other countries many CAM therapies are available over the counter and/or are often paid for out-of-pocket. The lack of administrative claims data on CAM therapies in countries where these costs are not covered or reimbursed means that cost studies require primary data collection (eg, patient self-report instruments) [100]. In their study on manual therapy for neck pain, Korthals-de Bos and colleagues used weekly cost diaries to obtain economic outcomes [27]. The second, related challenge is that many over-the-counter products, such as certain botanical medicines and nutritional supplements, are not standardized and of inconsistent quality. Standardization and quality will affect both the costs of the therapy and its outcomes. Finally, since there is often no provider "gatekeeper" controlling access to CAM therapies, monitoring of patient use can be complicated and labor intensive.

### Recommendations for future research

Despite the challenges described for economic evaluations of CAM therapies, these studies ought to be done. Every planned trial of CAM therapies should at least consider the feasibility of including an evaluation of economic impacts. Observational studies should also include these data, and as information accumulates regarding economic impacts, these costs and cost savings can be estimated more accurately. Although in the ideal every cost category shown in Table 2 should be measured and outcomes should include a measure of quality-adjusted life-years, the estimation of direct medical costs and savings associated with the therapy (eg, practitioner fees, lab fees, and the cost of herbs or other supplements prescribed) will be fairly straightforward for most studies, and the planned primary outcome of the study can serve as the measure of effects to determine cost effectiveness. Even if the clinical outcomes of a CAM therapy are similar or slightly less beneficial than those of usual care, a lower cost of care can still make these therapies attractive to decision makers. However, if no cost data are available, even highly effective therapies can be easily overlooked.

### Limitations

The limitations of this study are similar to those of the other reviews. First, the reader was not blinded to journals and article authors, which may have influenced results. Second, our measures of study quality depend on the information reported in an article, and no attempt was made to judge the merits of clinical or modeling assumptions made in the analyses. Third, only one reader read all the papers and extracted all the data. This may have lead to inaccurate reporting of results, and/or a biased interpretation of study quality. To maximize accuracy, data extraction was performed at least twice for each paper with several months break between extractions. Also, the approach and assumptions used to determine study quality were discussed at length with the other authors. These discussions led to a homogeneous approach being taken to both the application of the reporting quality criteria and the definition as to what constitutes an economic evaluation.

## Conclusion

As health care costs continue to rise, decision makers must allocate their increasingly scarce resources toward therapies which offer the most benefit per unit of cost. Economic evaluations inform evidence-based clinical practice and health policy. To be considered by these decision makers, CAM therapies and their outcomes must be known and compared to conventional approaches. However, CAM practitioners must themselves decide whether the cost of performing these studies is worth the potential impacts to their profession of being considered in managed care. Nevertheless, these evaluations will be done and they will be better done with practitioner involvement. Whereas the number and quality of these studies has increased in recent years and more CAM therapies have been shown to be good value, there are still not enough studies to measure the cost effectiveness of the majority of CAM. If CAM providers wish to increase the provision of therapies to improve population health, they must report the potential outcomes of CAM therapies widely and well.

## Competing interests

The author(s) declare that they have no competing interests.

## Authors' contributions

PH had the main responsibility for the manuscript and for bringing together the concepts of CAM and economics. PH also read and evaluated the quality of all papers included in the review. BC ensured that the health economic concepts were presented appropriately. PH and OC conceived the idea for the paper. OC contributed clinical and methodological insights. All authors read and approved the final manuscript.

## Pre-publication history

The pre-publication history for this paper can be accessed here:



## Supplementary Material

Additional File 1 Descriptions of included studies ordered by complementary and alternative medicine (CAM) modality, form of economic evaluation, and publication date. The appendix contains a table summarizing each of the 56 economic evaluations found in the systematic review. For each evaluation the following are reported: therapies compared, study population, study design and sample size, whether it was a full or partial economic evaluation, form of the evaluation, perspective, and summary results. Click here for file
