# Supplementary material for: Is complementary and alternative medicine (CAM) cost-effective? a systematic review
Source: BMC Complement Altern Med. 2005 Jun 2;5:11. doi: 10.1186/1472-6882-5-11 (PMC1182346; doi:10.1186/1472-6882-5-11)
Supplement: Additional File 1 — Descriptions of included studies ordered by complementary and alternative medicine (CAM) modality, form of economic evaluation, and publication date. The appendix contains a table summarizing each of the 56 economic evaluations found in the systematic review. For each evaluation the following are reported: therapies compared, study population, study design and sample size, whether it was a full or partial economic evaluation, form of the evaluation, perspective, and summary results. [file 1472-6882-5-11-S1.doc]

Appendix. Descriptions of included studies ordered by complementary and alternative medicine (CAM) modality, form of economic evaluation, and publication date

| Reference | Treatments Examined* | Condition/Group/ Cost Category | Study Design | Form of Economic Analysis, Perspective, and Results |
| --- | --- | --- | --- | --- |
| **Acupuncture** | | | | |
| Yeh et al, 2003[31] | Acupuncturists  Chiropractors | Patients seeing either type of practitioner | Consecutive visits to a random sample of acupuncturists (n=133, n=2561 visits) and chiropractors (n=130, n=2550 visits) | *Partial economic evaluation – cost analysis*  Examined the determinants of visit length for these practitioners. Mean visit length for acupuncturists was 56.6 minutes and were significantly longer if other adjunctive Asian therapies were used (e.g., cupping, magnets) or if self paid. Mean visit length for chiropractors was 21.5 minutes and was significantly longer if preventive counseling was included or if manual (versus instrument) spinal manipulation, soft tissue techniques and physiotherapeutics were used. |
| Liguori et al, 2000[32] | Acupuncture  Conventional care | Patients with migraine without aura | Randomized controlled multicenter trial (n=120) | Cost effectiveness (called cost benefit in the study)  National Health Service and societal costs  Total symptom scores dropped (improved) from 9823 to 1590 after 12 months in the acupuncture group (30 treatments over 4 months) and from 8405 to 3084 in the conventional care group. Total 12-month NHS and societal costs were -540,803 and 186,677,157 Lira, respectively, for the acupuncture group and 24,590,744 and 266,614,244 Lira, respectively, for the conventional care group. |
| Cherkin et al, 2001[33] | Acupuncture  Massage  Self-care education | Patients with chronic low back pain | Prospective, randomized comparison trial (n=262) | *Cost effectiveness*  *Health maintenance organization perspective*  After 1 year, the total cost of services related to back care were lowest for the massage group ($139, 1998 US$), next lowest for self-care ($200) and highest for acupuncture ($252). Cost differences were not significant. Symptom bothersomeness and disability were lowest at 1 year for massage (significantly lower than acupuncture), and next lowest for self-care. |
| Paterson et al, 2003[34] | Acupuncture  Homeopathy  Conventional care | Patients with dyspepsia | Prospective randomized controlled pilot (n=60) | *Cost effectiveness*  National Health Service perspective  Unique study design in that patients were asked their preferences for homeopathy or acupuncture and then either randomized to their preference or to control. Acupuncture and homeopathy both had lower scores on the primary outcomes (general health and well-being measures) at 6 weeks than controls. Acupuncture had higher total primary care costs at 6 months (£85, 2002) and homeopathy had lower costs (£60) than controls (£73). However, none of these results were statistically significant. |
| Wonderling et al, 2004[35] | Acupuncture  Conventional care | Patients with chronic headache | Prospective randomized controlled trial (n=401) | Cost utility (called cost effectiveness in study)  National Health Service (NHS) and societal perspectives  Total 1 year NHS and societal costs were significantly higher for the acupuncture group (£290 and £403, respectively, per patient for acupuncture versus £89 and £217 for conventional care, 2003), but QALYs were also slightly and significantly higher. |
| **Homeopathy** | | | | |
| van Haselen et al, 1999[36] | Homeopathic care | Patients with rheumatoid arthritis | Retrospective computerized record review and staff interviews (n=89) | Partial economic evaluation – cost description  Total costs of treating this sample of patients was £7632 of which £543 was assumed to be fixed. Consultation time with the doctor and dietician contributed 29% and homeopathic remedies and dietary supplements contributed 22% to total variable costs. |
| Frei and Thurneysen, 2001[37] | Homeopathic care  Conventional care | Children with acute otitis media | Prospective observational study on consecutive patients (n=230) | Partial economic evaluation – combined cost analysis and cost-outcome description (Called cost effectiveness in study)  Pain control was achieved in 72% of homeopathic patients by 12 hours and the remainder were put on antibiotics. Treatment costs were calculated by comparing actual homeopathic remedy and antibiotic costs (94.60 Swiss Francs) against the estimated costs of 100% antibiotic use (109.50 Swiss Francs) for a 14% savings. |
| Frenkel and Hermoni, 2002[23] | Complementary homeopathic remedies  Conventional medications | Patients with atopic and allergic disorders | Retrospective computerized medical chart review (n=48) | *Partial economic evaluation – cost analysis*  Computerized medication chart was analyzed for each subject to determine atopy-related prescription and non-prescription medication consumption 3 months before and after a homeopathic intervention. Found an average significant drop in medication costs for 3-months of 60% or $24 (1998 US$). |
| Jain, 2003[38] | Homeopathic remedies  Conventional medications | Homeopathically treated patients in one general practice | Retrospective review of medication records of consecutive patients (n=100) | *Partial economic evaluation – cost analysis*  Total cost of homeopathic medicines prescribed during the 4 year period was compared to the cost of conventional drugs that “would have been used by the same GP.” 64 out of 100 had a complete cure of symptoms, an additional 16 had “significant improvement,” and 10 are continuing treatment. For the 90 who completed treatment, conventional treatment was available for 84 and average drug cost savings were £60.40 (range of £12.48 increase to £703.95 decrease, 2002). |
| Trichard et al, 2003[39] | Homeopathy | Patients seen in homeopathic general practice | Cross-sectional survey data (n=149) | *Partial economic evaluation – cost description*  The diagnoses and treatments prescribed by a representative sample of homeopathic general practitioners in patient visits during 3 days in each of 3 seasons were recorded. Average cost of treatment for the 23 most common diagnoses (n=2216 patients) was 6.78€ of which 3.78€ was reimbursable. |
| Becker-Witt et al, 2003[40] | Homeopathic care  Conventional care | Patients with chronic diseases | Prospective, multicenter cohort study (n=493) | *Cost effectiveness*  *Health insurance company perspective*  Children treated with homeopathy improved significantly compared to those treated conventionally both according to patient and physician assessment. Adults improved significantly by self-assessment, but were statistically similar by physician assessment. No significant differences in costs were observed. |
| Paterson et al, 2003[34] |  |  |  | See listing under acupuncture. |
| Trichard and Chaufferin, 2003[41] | Homeopathic care  Conventional care | Recurrent acute rhino-pharyngitis in 18-month-old to 4-year-old children | Prospective, pragmatic observational study of two independent cohorts (n=499) | Cost effectiveness  *French National Insurance System, societal, and individual perspectives*  Found significantly fewer episodes of acute rhinopharyngitis, significantly fewer complications and a significantly better quality of family life in homeopathy cohort. Direct medical costs were statistically equivalent for the two groups, but there were significantly fewer sick-leave days in the homeopathy cohort. |
| Slade et al, 2004[42] | Homeopathic care  Conventional care | Patients seen by homeopathic physicians | Retrospective record review and pre/post survey of consecutive patients (n=97) | *Cost effectiveness*  *National Health Service perspective*  Primary symptoms improved significantly (2.49 on a 6 point scale) in the 6 months after homeopathy. Numbers of physician visits decreased significantly for the 6 months after homeopathy as compared to the 6 months before by 1.18 visits. Estimated total annual medication cost savings for the patients that answered this questionnaire (n=49) were £2807. |
| Van Wassenhoven and Ives, 2004[43] | Homeopathic care  Conventional care | Patients seen by homeopathic physicians | Cross-sectional survey of homeopathic physicians (n=80) and their patients (n=782) | *Cost effectiveness*  *Individual perspective*  Patients seen on one specified day “estimated their average annual expenditure on consultations” before (370€) and after (287€) initiating homeopathy. Homeopathic physicians who provided detailed data on conventional drugs prescribed (n=47) had a total drug expenditure of approximately one-third that of conventional colleagues. 89% of patients reported that homeopathy had improved their physical condition. |
| Trichard and Chaufferin, 2003[44] | Homeopathic care  Conventional care | Patients with anxiety disorders | Prospective, pragmatic observational study of two independent cohorts (n=394) | Cost effectiveness and cost utility  French National Insurance System and societal perspectives  Equivalent statistical results for both cohorts in terms of medical effectiveness, utility, and overall cost reimbursed by National Insurance System, with significantly fewer sick-leave days. |
| **Manual Therapy (Chiropractic, Spinal Manipulation, Bone Setting, Massage)** | | | | |
| Hess and Mootz, 1999[45] | Chiropractic care  Conventional and osteopathic care | Hypothetical patients in a variety of clinical vignettes | Cross-sectional national random sample of chiropractors (n=127) compared to previous estimates for conventional and osteopathic practitioners | *Partial economic evaluation – cost analysis*  Vignettes were developed representative of commonly seen cases and including vignettes included in the Harvard national surveys of conventional and osteopathic practitioners. In the survey, chiropractors were asked estimate relative work compared to a base case for various evaluation/management, radiology, and spinal manipulation tasks. Chiropractors were found to perform similar total work for evaluation/management and increased work for radiology interpretation than conventional and osteopathic practitioners. Osteopathic work for manipulation was higher than for chiropractors. |
| Yeh et al, 2003[31] |  |  |  | See listing under acupuncture |
| Burton et al, 2000[46] | Spinal manipulation  Conventional care | Patients with symptomatic lumbar disc herniation | Prospective randomized controlled trial (n=40) | *Cost effectiveness*  *Institutional perspective*  Leg and back pain and disability improved significantly more in the manipulation group for the first few weeks, and significantly improved in both groups by 12 months, but there was no significant differences between groups by 12 months. Cost analysis showed manipulation to save £300 per patient over the year. |
| Cherkin et al, 2001[33] |  |  |  | See listing under acupuncture. |
| Hemmila, 2002[47] | Bone setting (traditional soft tissue, spinal, and joint manipulation)  Physiotherapy  Light exercise therapy | Patients with back pain | Retrospective cost analysis pre-intervention then prospective randomized controlled trial (n=108) | *Cost effectiveness*  *Societal perspective*  Most quality of life subscales were significantly improved one year after intervention, but only the emotional reactions subscale differed significantly between groups—bone setting was better than exercise. The use of health care remained unchanged between the year before and year after the intervention by group with bone setting having the highest total costs related to back pain (FIM10792, 1994) and physiotherapy having the lowest costs (FIM3236). |
| Stano et al, 2002[48] | Chiropractic care  Conventional care | Patients with acute or chronic ambulatory low back pain | Prospective practice-based observational study (n=2263) | *Cost effectiveness*  *Institutional perspective*  Improvement in pain and in disability per dollar cost were similar between patients seen at chiropractic clinics and patients seen at conventional medical clinics. Annual treatment costs were significantly higher in patients seen at chiropractic clinics ($214, 1995 US$) as compared to conventional medical clinics ($123). |
| Korthals-de Bos et al, 2003[27] | Manual therapy  Physiotherapy  Conventional care | Patients with neck pain | Prospective randomized controlled trial using cost diaries and clinical outcomes (n=183) | Cost effectiveness and cost utility  Societal perspective  Manual therapy patients had a significantly higher recovery rate through 26 weeks, however, the difference across groups was no longer significant at 52 weeks. Manual therapy also had a significantly larger reduction in pain intensity than physiotherapy at 52 weeks, and a larger increase in utility, however this was not significant. Total one-year costs were the lowest for manual therapy ($402, US$), next lowest for physiotherapy ($1167), and highest for general practitioner care ($1241). |
| **Spa Therapy** | | | | |
| Shani et al, 1999[49] | Climatotherapy (combination of mineral salts, air and resulting mild UV)  Other modalities | Patients with psoriasis | Cost estimates applied to previous effectiveness studies across a range of therapies | Cost effectiveness  *Institutional perspective*  Cost per one week remission were estimated for climatotherapy as approximately $500 (US$) which was comparable to the costs of topical corticosteroids and methotrexate therapy, the other lowest-cost alternatives. |
| Brefel-Courbon et al, 2003[50] | Spa therapy  Conventional care | Patients with Parkinson’s disease | Prospective randomized cross-over controlled trial (n=31) | *Cost effectiveness*  Health care payer’s perspective  At 4 weeks spa therapy significantly improved stigma, communication, physical and mental health, and psychological distress, but at week 20 no differences remained. The mean direct medical cost over 20 weeks was significantly reduced in the spa therapy period from 1328€ (2002) to 1380€. |
| Van Tubergen et al, 2002[51] | Combined spa-exercise therapy (at two different spas)  Conventional care | Patients with ankylosing spondylitis | Prospective randomized controlled trial (n=120) | Cost effectiveness and cost utility  Societal perspective  There was a significant improvement in functional ability and in utility in the two spa groups (two different spas) as compared to the control group. The mean total costs of therapy were 3023€ (2001) and 3240€ for the two spa groups, significantly higher than the 1754€ for the control group. The incremental cost-effectiveness ratios for the two spa groups each compared to control were 7465€ and 18565€ per quality-adjusted life year (QALY). |
| **Mind-Body/Stress** | | | | |
| Roth and Stanley, 2002[52] | Complementary mindfulness-based stress reduction  Conventional care | Inner-city patients | Retrospective medical chart review pre-/post-intervention (n=47) | *Partial economic evaluation – cost analysis*  There was a significant decrease in chronic care visits from the one year before the 8-week mindfulness-based stress reduction course to the year after. There was also a significant decrease in total medical visits in the 36 patients completing the Spanish course. |
| Herron and Hillis, 2000[24] | Complementary transcendental meditation  Conventional care | Persons in the Quebec health care system | Retrospective controlled pre-/ post-intervention study (n=2836) | Cost minimization  *National health insurance perspective*  Yearly rate of increase in government payments to physicians was similar between groups before transcendental meditation (TM) practice was initiated. After TM, the TM group’s payments dropped 1-2% per year and the control group’s costs increased up to 11.73% annually. This difference was significant. |
| Tusek et al, 1999[53] | Complementary guided imagery via tape  Conventional postoperative care | Cardiac surgery patients | Prospective, unmasked randomized controlled trial (n=100) | Cost effectiveness  *Institutional perspective*  The guided imagery group experienced a significantly smaller increase in pain and anxiety day 1 post-operation compared to baseline than the control group, and their hospital stay was significantly (2 days) shorter for a 19% savings in direct costs. |
| van Dixhoorn and Duivenvoorden, 1999[54] | Complementary relaxation therapy  Exercise training | Patients with previous myocardial infarction | Prospective randomized controlled trial (n=156) | Cost effectiveness  *Institutional perspective*  The adjusted odds ratio for cardiac events for relaxation therapy was 0.52 and significant. The average readmission rate decreased by 31% and the average number of hospital days decreased by 30% in the relaxation group. The average cost of readmissions dropped more (46%) due to a reduction in cardiac surgery. The medical cost savings more than offset the extra cost of the relaxation training. |
| Jacobsen et al, 2002[55] | Complementary professionally administered stress management  Complementary self-administered stress management  Conventional psychosocial care | Cancer patients undergoing chemotherapy | Prospective randomized controlled trial (n=411) | Cost effectiveness  Payer and societal perspectives  Self-administered stress management training (SSMT) was found to have better physical functioning, greater vitality, fewer role limitations from emotional problems, and improved mental health while professionally administered stress management (PSMT) was found to not differ in effectiveness from usual care. The average incremental costs of PSMT per patient over usual care were $110 (2000 US$) from the payer persepective and $136 from the societal perspective. SSMT had significantly lower average incremental costs of $47 from the payer perspective and $73 from the societal perspective. |
| Mehl-Madrona, 2002[56] | Traditional Chinese medicine, body therapy, and guided imagery  Conventional care | Patients with uterine fibroids | Prospective study with retrospective chart review for matched controls (n=74) | *Cost effectiveness*  *Institutional perspective*  Fibroids shrank or stopped growing in significantly more of the treatment group (22/37) than of the matched controls (3/37). Cost of care for the treatment group averaged $3800 (US$) and was “significantly greater than costs for the comparison group.” |
| Bittman et al, 2003[57] | Group recreational music making  No intervention | Long-term care workers | Prospective randomized controlled cross-over trial with a model for costs (n=112) | Cost effectiveness  *Long-term care facility perspective*  Burnout and mood indicators significantly improved while workers were participating in the 6-week intervention. Costs were projected using a model based on nursing home staff satisfaction and turnover rates, projected annual cost savings were estimated at $89,100 (US$) for a typical 100-bed facility. |
| **Hypnosis** | | | | |
| Lang and Rosen, 2002[58] | Complementary hypnosis  Conventional IV conscious sedation | Patients undergoing interventional radiologic procedures | Retrospective cost data and decision analysis model on randomized trial results (n=79) | Partial economic evaluation – cost analysis  *Institutional perspective*  The study did not report the effectiveness findings of the randomized trial, but the costs and probabilities of over-, under-, or appropriate sedation from the trial were used to build a decision analysis model. Direct costs for adjunct hypnosis were $300 per patient (US$ 2000) and $638 for standard sedation. |
| Meurisse et al, 1999[59] | Hypnosis with conscious sedation  General anaesthesia | Patients undergoing cervical endocrine surgery | Prospective study of treatment (n=118) and matched controls (n=121) | *Cost effectiveness*  *Institutional perspective*  Patients receiving hypnosis reported significantly less post-operative pain and fatigue, needed significantly fewer pain medications, and were significantly more satisfied than the control group. Their hospital stay was significantly shorter (44 versus 74 hours), and their return to normal activity was significantly faster (12 versus 33 days). |
| Defechereux et al, 1999[60] | Hypnosis with conscious sedation  General anaesthesia | Patients undergoing cervical endocrine surgery | Prospective study of treatment (n=218) and matched controls (n=119) | *Cost effectiveness*  *Institutional perspective*  Patients receiving hypnosis reported significantly less post-operative pain and fatigue, needed significantly fewer pain medications, and were significantly more satisfied than the control group. Their hospital stay was significantly shorter (44 versus 74 hours), and their return to normal activity was significantly faster (12 versus 33 days). |
| **Botanical Medicine** | | | | |
| Chrubasik et al, 2001[61] | Complementary proprietary willow bark extract at 2 doses  Conventional orthopedic therapy | Patients with low back pain | Prospective open nonrandomized study (n=451) | *Cost effectiveness*  Social and private health insurance company perspectives  At four weeks 40% of the high dose willow bark group were pain free compared to 19% of those in the low dose group, and 18% of those in the conventional therapy group, a significant difference across groups. Average social (private) total costs per patient also were significantly different between groups: 71.9 (96.3) DM for high dose willow bark, 56.3 (75.7) DM for low dose and 85.9 (161.6) for conventional therapy. |
| Stothers, 2002[62] | Concentrated cranberry concentrate tablets  Unsweetened cranberry juice  Placebo | Women with history of urinary tract infection | Prospective randomized controlled trial followed by stochastic decision tree modeling (n=150) | Cost effectiveness  Societal perspective  Both cranberry juice and tablets significantly decreased the number of women experiencing at least one symptomatic urinary tract infection (UTI) during the year compared to placebo. The cost-effectiveness ratio for juice over placebo was $3333 per UTI prevented, and for tablets it was $1890 per UTI prevented (CN$). |
| **Nutritional Supplements** | | | | |
| Shabert et al, 2000[63] | Glutamine-antioxidant supplementation  Recombinant human growth hormone | Patients with human immunodeficiency virus (HIV) infection | Retrospective cost analysis based on results of a randomized controlled trial (n=21) | *Partial economic evaluation – cost analysis*  The cost for a gain of 1.0 kg in body cell mass using recombinant human growth hormone is estimated at $9230 (US$) versus $220 for glutamine-antioxidant supplementation. |
| Blakeley and Ribeiro, 2002[64] | Glucosamine | Older adults | Cross sectional survey (n=65) | *Partial economic evaluation – cost description*  The amount spent per maonth on glucosamine products ranged from less than $10 (CN$) to $50. The majority (69%) paid $20 or less per month. |
| Chang et al, 2003[65] | Antioxidants and zinc | Age-related macular degeneration (AMD) | Cross sectional survey of patients followed by cost analysis (n=108) | *Partial economic evaluation – cost description*  The survey revealed that although 79% were taking supplements, only 68% were taking at least one nutrient found to be helpful in the Age-Related Eye Disease Study (beta-carotene, vitamin C, vitamin E, and zinc), and none met the recommended doses of all four nutrients. Analysis across available combination and individual nutrient supplements determined that full recommended doses could be achieved at a minimum cost of $0.52 (CN$). |
| Senkal et al, 1999[66] | Complementary immunonutrition (arginine, n-3 fatty acids, RNA)  Conventional pre/post operative diet | Patients undergoing elective upper gastrointestinal surgery | Prospective randomized double-blind multicenter controlled clinical trial (n=154) | Cost effectiveness  Hospital perspective  Treatment group received immunnonutrition 5 days prior to surgery and 10 days after. The treatment group experienced significantly fewer infectious complications, and total complications than the control group. The costs per complication-free patient were 1504 DM (1998) for the treatment group and 3587 DM for the control group. |
| Franzosi et al, 2001[25] | Complementary omega-3 polyunsaturated fatty acids  Conventional care | Patients with recent myocardial infarction | Prospective follow up to a randomized controlled open-label trial (n=5664) | Cost effectiveness  Third-party payer perspective  The incremental cost-effectiveness ratio for patients taking omega-3 fatty acids versus regular care was 24603€ (1999) per life-year saved (95% confidence interval: 22646 to 26930). |
| Zhou et al, 2003[67] | Complementary glutamine  Conventional enteral formula | Severe burn patients | Prospective, double-blind, randomized controlled trial (n=40) | *Cost effectiveness*  *Institutional perspective*  Both groups received enteral nutrition for 12 days after burns with one group receiving glutamine in addition to the standard enteral formula. The glutamine group had significantly lower intestinal permeability, fewer infections, and better weight maintenance. Total hospitalization costs were significantly lower in the glutamine group ($749, US$) more than offsetting the increased costs of glutamine ($222). |
| Smedley et al, 2004[68] | Complementary preoperative, postoperative, or both oral nutritional supplementation  Usual pre- and postoperative care | Patients undergoing lower gastrointestinal tract surgery | Prospective randomized controlled trial (n=152) | *Cost effectiveness*  *Hospital perspective*  There was significantly less postoperative weight loss in the group that got both pre- and postoperation supplementation (SS) than in the control (CC) and postoperation supplementation only (CS) groups, and significantly fewer minor complications in the SS and CS groups than in the CC group. Average total costs over 4 weeks were highest in the CC group, but this was not significant. |
| **Diet** | | | | |
| Gilbert et al, 1999[69] | Complementary ketogenic diet  Conventional care | Children with difficult to control epilepsy | Retrospective/ prospective study of consecutive children (n=85) | *Partial economic evaluation – cost analysis*  Medication costs were collected prospectively for 1 year after children were put on the diet, and the previous year’s medication costs were estimated based upon medication use at study start. Total costs of medications were estimated as $116980 (1997 US$) at study start and $38,603 after 1 year on the diet. Since medications were reduced gradually over the year, the actual cost of medications for the year after the study start was $45064. |
| MacCracken and Scalisi, 1999[70] | Ketogenic diet | Children with epilepsy | Retrospective review of patient records (n=11) | *Partial economic evaluation – cost description*  The study reports the cost of various components of the intervention, including its development, but does not compare these costs to pre-diet levels or to other interventions. |
| Mandel et al, 2002[71] | Complementary ketogenic diet  Conventional care | Children with intractable epilepsy | Retrospective patient database review of consecutive children (n=15) | *Partial economic evaluation – cost analysis*  Total medical center costs across the 15 children were $352,820 for the 6 to 12 month prediet period, $41,222 for diet initiation, and $149,437 for the 6 to 12 months post-diet initiation. |
| Goldhamer, 2002[72] | Water-only fasting  Conventional care | Patients with high-normal blood pressure | Retrospective before/after claims analysis (n=24) | *Cost effectiveness*  *Institutional perspective*  Participants’ blood pressure dropped an average of 30/11 mmHg and they lost 26 pounds on the program. Average annual medical care costs (including medications) dropped from $5784 per patient before the intervention to $3000 after (US$). The cost of the program was not included, but it was noted that it was less than the $2784 savings. |
| Raynor et al, 2002[73] | High nutrient-dense diet promoted through group and/or individual sessions  Conventional diet | Families with obese members | Prospective pre-/ post-intervention trial (n=20) | Cost effectiveness  *Individual perspective*  There were significant decreases in weight, energy intake, and intake of low-nutrient-dense foods at 6 months and 1 year from baseline. The daily dietary cost also decreased significantly from baseline to 1 year from $6.77 to $5.04 per meal per participant (US$), but did not change by cost per 1000 kcals. |
| Norris et al, 2004[74] | Potassium-rich diet  Potassium chloride pills | Postoperative cardiac patients | Prospective randomized controlled trial (n=38) | *Cost effectiveness*  *Hospital costs*  No significant difference between groups in serum potassium levels postoperatively. Length of hospital stay was significantly lower in the diet group (5.0 versus 6.3 days). |
| **Biofeedback** | | | | |
| Mellgren et al, 1999[75] | Biofeedback  Sphincteroplasty  Conservative nonoperative treatment | Patients with fecal incontinence | Retrospective cost study of consecutive patients (n=63) | *Cost effectiveness*  *Institutional perspective*  The study was intended to document the long-term direct medical costs of fecal incontinence from obstetric injuries at one clinic, but also broke down costs and results by type of treatment. There was a significant improvement in incontinence score after surgery, but none from biofeedback or conservative treatment. Estimated annual costs per patient decreased for surgery and conservative treatment ($242 to $134 and $179 to $147, 1996 US$) and increased for biofeedback ($216 to $261). |
| Ryan and Gevirtz, 2004[76] | Biofeedback-based psychophysiological treatment  Conventional care | Patients with “functional” disorders (irritable bowel syndrome, fibromyalgia, functional cardiac pain, myofascial pain, panic or anxiety with somatic) | Prospective randomized controlled trial (n=70) | *Cost effectiveness*  *Institutional perspective*  Symptom severity decreased significantly by week 8 for the treatment group who completed the program. Monthly medical costs dropped between the 6 months before and 6 months after the treatment period more in the treatment group participants that completed the program (n=19) versus the control group ($72 versus $9, US$). However, monthly medical costs for treatment dropouts increased by $134. |
| **Hyperbaric Oxygen Therapy** | | | | |
| Abidia et al, 2003[77] | Hyperbaric oxygen  Hyperbaric air | Diabetic patients with ischemic lower-extremity ulcers | Prospective randomized controlled trial (n=16) | *Cost effectiveness*  *Institutional perspective*  Significantly more patients had their ulcers healed in the oxygen group than in the control group (5/8 versus 0/8) at one year. The oxygen group had a significantly lower total annual cost for ulcer dressing than the control group (£1972 versus £7942). This difference more than covered the £3000 cost of the hyperbaric oxygen treatment. |
| Guo et al, 2003[78] | Complementary hyperbaric oxygen therapy  Conventional care | Patients with severe diabetic foot ulcers | Decision tree analysis based upon previous studies (n=1000) | Cost utility (Called cost effectiveness in the study)  *Institutional perspective*  Incremental costs per additional quality-adjusted life-year (QALY) was estimated as $27,310 (US$) after one year, $5,166 after five years, and $2,255 after twelve years. |
| **Miscellaneous** |  |  |  |  |
| Burns et al, 2000[79] | Conventional aromatherapy | Intrapartum midwifery patients | Prospective assessment of treatment group (n=8058) | *Partial economic evaluation – cost-outcome description*  More than 50% of the mothers found aromatherapy helpful. Total cost of aromatherapy for one year’s group of 1592 mothers was $0.80 per woman (1997 US$). |
| Dobson et al, 2004[80] | Daily multivitamin  Present multivitamin use | Older adults | Estimate of five-year potential savings based on results of other studies | *Partial economic evaluation – cost analysis*  Estimated savings to Medicare beneficiary population (>65 years) as the benefits from reductions in hospital, skilled nursing facility, and home health payments from reduced heart disease and infections from the use of multivitamins minus the cost of multivitamins to that population likely to need them. Total savings of $1.6B (US$) were estimated. Estimates were based on evidence in the literature with an emphasis on meta-analyses and randomized controlled trials. |
| Kail, 2001[81] | Electrodermal screening  Conventional care | Patients with allergy/sensitivity | Retrospective pre-/ post-intervention study of consecutive patients (n=90) | Cost effectiveness  *Institutional perspective*  The severity of allergy symptoms decreased significantly after the intervention, and the average total costs of treatment (visits, testing, support supplements, treatment) was $822 (US$) per patient. |
| Larsen et al, 2002[82] | Complementary  custom-made biomechanic shoe orthoses  Usual footwear | Recent military conscripts | Prospective randomized controlled trial (n=146) | *Cost effectiveness*  *Military perspective*  The intervention group had significantly fewer back or lower extremity problems during the 3 month study period. They also had significantly fewer days off duty. Costs to prevent were estimated to be $98 (US$) per back or lower extremitiy problem prevented, and $3750 per day off duty prevented. |

US$ = United States dollars; CN$ = Canada dollars; £ = United Kingdom pounds; € = Euros; DM = German Deutsche marks; FIM = Finland Markkaa; if not stated in the study, currency year was estimated as one year prior to publication.

* The term “complementary” in this column indicates that those therapies are offered in addition to the last therapy listed.

*Italics* type for the form of economic evaluation or perspective indicates that the reported data were inferred from the information in the study and not stated explicitly. A year is given for the monetary figures only if it was reported in the study. If statistical significance testing was applied to the results reported this is indicated. Otherwise, no significance tests were performed in the study for the results reported here. QALY = quality-adjusted life-year.
